# Head and Neck Merkel Cell Carcinoma: A 12-Year Single Institutional Experience

**DOI:** 10.1016/j.jpra.2022.05.005

**Published:** 2022-05-14

**Authors:** C.M. Hurley, D. ALNafisee, D. Jones, J.L. Kelly, P.J. Regan, A.J. Hussey, N. McInerney

**Affiliations:** 1 Department of Plastic & Reconstructive Surgery, University Hospital Galway, Galway, Ireland; 2 Royal College of Surgeons in Ireland, St. Stephen's Green, Dublin, Ireland

**Keywords:** Merkel cell, Head and neck, Neuroendocrine, Sentinel node, Radiotherapy

## Abstract

**Background:**

Merkel cell carcinoma (MCC) is an aggressive malignancy of presumed neuroendocrine origin. Most case series of MCC are limited by low case numbers and are not specific to head and neck tumours. The purpose of this study was to provide a focused review of head and neck MCC diagnosis and management in a single Irish institution.

**Methods:**

Patient's demographics, tumour characteristics, pathological diagnosis, surgical treatment, adjuvant treatment, subsequent management and clinical course were collected. Estimates of progression-free MCC survival rates were calculated by the Kaplan–Meier statistical model. A Pearson product-moment correlation coefficient examined the association between surgical margins and disease-free follow-up.

**Results:**

In total, 11 patients were treated for head and neck MCC with a mean age of 79.6 years (range = 69–91 years). The mean average follow-up duration of patients was 18.3 months. Of the cohort, 18% (*n*=2) had a sentinel node biopsy (SLNB). A selective neck dissection was subsequently performed in 18% (*n*=2). In total, 72% (*n*=8) of patients received adjuvant radiotherapy. Median disease-specific survival was 15 months for the SLNB group and 17 months for the non-SLNB group, not statistically significant (*p*=0.23). There was no significant association between surgical margins and disease-free follow (*p*=0.65).

**Conclusions:**

Our case series adds to a limited body of evidence of head and neck MCC. Surgery remains the treatment priority in localized disease, with an increasing role of SLNB for accurate prognostication and staging. Early management of stage I disease results in moderate long-term disease-free survivability.

## Introduction

Merkel cell carcinoma (MCC), also termed *cutaneous neuroendocrine carcinoma*, is a rare yet aggressive tumour of neuroendocrine cell origin that commonly presents in the head and neck region.^1^ First described in 1972, it is characterized by a high incidence of local recurrence and regional and distant metastasis.^2^ Despite its increasing incidence, MCC remains atypical and, as a result, poorly characterized.^3^ The diagnosis of MCC is rarely clinically suspected because the primary tumour often lacks predictable characteristics and is often asymtomatic.^4^ The primary manifestation for the disease includes a rapidly growing, red or purple painless nodule.^5^ Increasing age, immunosuppression, ultraviolet light, male sex and the Merkel cell polyomavirus are independent risk factors for developing MCC.^1^^,^^6^ The current management of stage I-II disease includes surgical excision with wide margins with adjuvant post-operative radiotherapy in most cases.^4^^,^^5^

The role of sentinel node biopsy (SLNB) in the management of MCC remains unclear. Evidence suggests that SLNB negativity is a strong predictor of longer disease-free survival and overall survival.^7^^,^^8^ Conversely, others suggest no prognostic value of SLNB.^3^^,^^9^ MCC of the head and neck may require distinct review from MCC in other anatomic locations.^3^ Head and neck MCCs are complicated by complex draining patterns and aggressive tumour characteristics.^3^, ^4^, ^5^^,^^10^^,^^11^ Nonetheless, the current MCC management guideline from the National Comprehensive Cancer Network (NCCN) recommends a diagnostic SLNB for all clinically node-negative patients who are fit for surgery.^4^

Most case series of MCC are limited by low case numbers and are not specific to head and neck tumours. The purpose of this study was to provide a focused review of head and neck MCC diagnosis and management in a single Irish institution.

## Methods

### Patient selection

The study was approved by our local institutional ethics review committee. All head and neck MCC patients from 2008 to 2020 were retrospectively identified via the ‘Hospital Inpatient Enquiry Department’ system, a prospectively maintained coded database of patient diagnosis. This was cross-referenced with the institutions’ ‘Tumour Database’ histopathological archive system. Any diagnosis of MCC above the clavicle was included. All patient data were collected and stored anonymously in an encrypted database in Microsoft Excel (Microsoft Corp., Redmond, WA, USA). The patient's demographics, tumour characteristics, pathological diagnosis, surgical treatment, adjuvant treatment, subsequent management and clinical course were collected. Tumours were staged via the latest American Joint Committee on Cancer (AJCC) system.^12^

### Statistics

Statistical analysis was carried out using SPSS version 18 (SPSS Inc., Chicago, IL, USA), with alpha values <0.05 indicating statistical significance. Estimates of progression-free MCC survival rates were calculated by the Kaplan–Meier statistical model. The progression of disease was defined as the regional or metastatic spread of MCC. A Pearson product-moment correlation coefficient was conducted to examine the association between surgical margins and disease-free follow-up.

## Results

### Patient and Tumour Characteristics

Between 2008 and 2020, eleven patients were treated for head and neck MCC in our institution. Of these, eight were male, and three were female. The mean age of the cohort was 79.6 years (range = 69–91 years). Six tumours were on the cheek, and one on the forehead, brow, upper eyelid, upper lip and scalp. The size of the tumours ranged from 5 mm to 20 mm in their widest diameter (range = 5mm–20mm). The mean average follow-up duration was 18.3 months (range = 3–72 months). In total, 36.4% (*n*=4) patients had a recurrence at a mean time of 12.25 months (range = 8–17 months). Of these, two patients developed distant metastatic MCC. The distribution of the primary lesions, patient-associated demographics and their management is listed in Table 1.

**Table 1:** Summary of patient characteristics.

Patient	Sex/age	Primary tumourlocation	Stage at presentation	Surgical treatment	Wider excisionmargin (cm)	Reconstruction	SLNBx	LND	Adjuvant therapy	Progression-free follow-up	Prognosis
1	M/78	Brow (L)	I	WLE	1.5	FTSG	No	No	Rad	13 months	No recurrence
2	M/82	Cheek (L)	I	WLE	2	Closure	No	No	Rad	8 months	Distant Metastasis
3	M/72	Cheek (L)	I	WLE	2	Closure	Yes	Yes	Rad + chemo	15 months	Distant metastasis
4	M/84	Upper eyelid (L)	I	WLE	2	Local flap	No	No	Rad + chemo	9 months	Regional recurrence
5	M/69	Forehead (R)	I	WLE	1	FTSG	No	No	No	46 months	Alive, NED
6	M/79	Cheek (R)	I	WLE	1	Closure	No	No	No	17 months	Local recurrence
7	F/79	Upper Lip	I	WLE	1	Closure	No	No	Rad	6 months	No recurrence
8	F/91	Cheek (L)	IIIB	WLE	2	Local flap	Yes	Yes	Rad + chemo	72 months	No recurrence
9	F/77	Cheek (L)	I	WLE	1	FTSG	No	No	Rad	3 months	No recurrence
10	M/85	Cheek (R)	I	WLE	1	Closure	No	No	No	6 months	No recurrence
11	M/84	Scalp	I	WLE	2	Local flap	No	No	Rad	7 months	No recurrence

Abbreviations: WLE = wide local excision; FTSG = full-thickness skin graft; SLNBx = sentinel lymph node biopsy; LND = lymph node dissection; Rad = radiation therapy; chemo = chemotherapy; NED = no evidence of disease.

### Surgical Treatment and Reconstruction

The index biopsy was performed by a plastic surgeon in 73% of cases (*n*=8). The other three cases were referred from general surgeons in a tertiary centre. Of these, 18% (*n*=2) were excisions, and 9% (*n*=1) was a punch biopsy. All patients had a further wider excision with a mean margin of 1.14 cm (mean = 0.6–2 cm). After wider excision, five were closed primarily, three with a local flap, and three with a full-thickness skin graft. A Pearson product-moment correlation coefficient revealed no significant association between surgical margins and disease-free follow-up (*p*=0.65).

### Regional Lymph Nodes

Of the cohort, 18% (*n*=2) had an SLNB. One node was identified in each SLNB. The mean size of the nodes was 8 mm in the widest diameter (range = 7–9 mm). Both of these were positive for MCC with H&E and CK-20 immunostaining. A selective neck dissection was subsequently performed in 18% (*n*=2). One demonstrated 18 nodes, 7 of which MCC was observed. Another had 15 nodes, 7 of which were positive for MCC. There were no associated complications with each SLNB.

### Adjuvant Radiotherapy

In total, 72% (*n*=8) of patients received adjuvant radiotherapy. Of these, 54% (*n*=6) had radiotherapy after the wider excision as adjuvant therapy. An additional 18% (*n*=2) had radiotherapy for treatment for recurrent disease. One patient died from unrelated illness awaiting adjuvant radiotherapy.

### Prognosis

**Figure 1:**
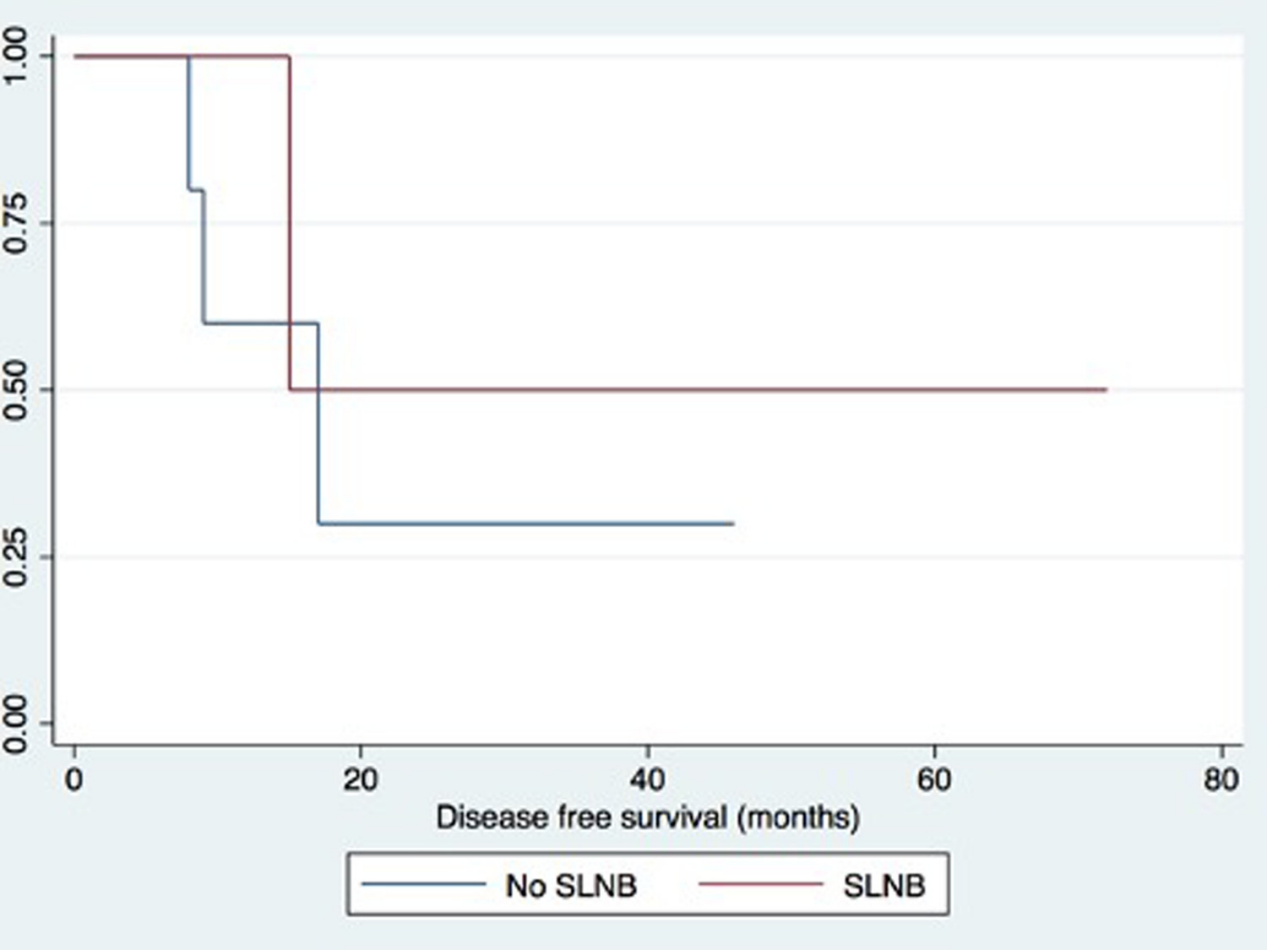
Kaplan–Meier curve demonstrating MCC-specific disease-free survival according to sentinel lymph node procedure.

The median disease-free follow-up for all patients was 17 months (range = 3–46 months). A Kaplan–Meier graph in Figure 1 summarizes the effect of SLNB on estimated disease-specific survival (DSS). SLNB had an effect on the DSS time compared to those that did not have an SLNB as part of primary management. The median DSS was 17 months. Median DDS was 15 months for the SLNB group and 17 months for the non-SLNB group, but this was not statistically significant (*p*=0.23). One patient developed local recurrence at the primary tumour scar, and one patient developed regional progression. Distant metastasis developed in two patients.

## Case Series

### Case 1

A 78-year-old Caucasian man had an excisional biopsy of a tan keratotic nodule on his left brow demonstrating MCC on a background history of malignant melanoma. The lesion measured 20 × 13.5 mm in size, and perineural and lymphovascular invasion was present. Peripheral and deep margins were clear. A follow-up wider excision of 1 cm was performed, and the defect was reconstructed with a full-thickness skin graft. He had radiotherapy to the surgical bed and is disease free for 13 months post-operative.

### Case 2

An 82-year-old gentleman had an excision of a pearly white lesion of his left cheek. The histopathology demonstrated a 14 mm MCC with perineural invasion with extensively positive deep margins. He had a further wider excision of 1 cm. Eight months later, he presented with recurrent local disease involving the left lower eyelid and medial canthus. This was resected and reconstructed with a full-thickness skin graft. He remained disease free for 8 months. Subsequent imaging demonstrated regional lymph node involvement and a 5 mm lung metastasis. He received 60 Gy of radiotherapy to the tumour site and regional lymph nodes. However, he died of metastatic disease 2 months after completing radiotherapy.

### Case 3

A 72-year-old gentleman was referred with a red, raised nodular lesion on his left cheek. Excisional biopsy demonstrated a 12 mm MCC with negative radial and deep margins. He had a subsequent wider excision of 2 cm. A sentinel lymph node biopsy of one 9 × 7 × 6 mm node was negative for metastatic disease. He had 54 Gy of radiotherapy delivered to the tumour bed. He was disease free, until he developed regional recurrence in the neck at 15 months post-operative. A CT scan demonstrated large lobulated masses in the left submandibular area indicative of regional lymph node involvement. A PET CT demonstrated no distant metastasis. He had a selective neck dissection which identified 18 lymph nodes, nine of which were positive for MCC. He had one cycle of cisplatin and oral etoposide followed by 50Gy of radiotherapy to the involved regional lymph nodes and is disease free to date.

### Case 4

An 84-year-old male was referred with a recurrence of an MCC of his left upper eyelid. A wider excision of 2 cm was performed, and the defect was reconstructed with a cervicofacial flap. She received 66 Gy of radiotherapy to the tumour site. A CT brain, thorax abdomen and pelvis identified no metastatic disease. However, she presented with metastatic disease 9 months later and was commenced on systemic carboplatin and oral etoposide. She died 11 months later due to complications of chronic lymphoid leukaemia.

### Case 5

A 60-year-old gentleman presented with a slow-growing ulcerating nodule on his right forehead in March 2016. An excisional biopsy demonstrated a fully excised 12 × 10 × 6 mm irregularly shaped MCC, and a wider excision of 1 cm was reconstructed with a full-thickness skin graft. He received no radiotherapy and is disease free for 46 months later.

### Case 6

A 79-year-old male was referred from a peripheral hospital with a MCC over the site of a previous basal cell carcinoma on his right cheek. A wider excision of 1 cm was performed to muscle and closed primarily. Following this, 17 months later, he presented with two subcutaneous nodules adjacent to the previous scar. A fine-needle aspiration of the nodules was positive for MCC. He was treated with radical radiation therapy and concomitant cisplatin and etoposide, which was discontinued due to his declining performance status. The patient died due to unrelated heart disease.

### Case 7

A 79-year-old female was referred with a 5 × 5 mm dark papule on her upper lip present for 8 months. An excisional biopsy demonstrated an MCC with negative peripheral and deep margins. A wider excision of 1 cm was performed and closed primarily. She is currently undergoing radiotherapy treatment to the tumour site.

### Case 8

A 91-year-old female presented with 15 × 14 mm MCC on her left. A wider excision of 2 cm was performed, and the defect was reconstructed with a local flap. Her investigative CT scan demonstrated probable metastatic disease in the left submandibular region, and she subsequently had a left neck SLNB. One 7 × 7 × 7 mm node was positive for MCC, and she had a completion lymphadenectomy. Post-operatively, she had 60 Gy to the tumour bed and regional lymph nodes and had two cycles of systemic cisplatin. At 6 years post-operative, she demonstrated no recurrence and died of an unrelated causes.

### Case 9

A 77-year-old female was referred from a tertiary hospital with a punch biopsy confirmed MCC of the left cheek. One month later, a wider excision of 1 cm was performed demonstrating a 20 × 17 mm MCC. She died of unrelated illness while waiting for scheduled radiotherapy.

### Case 10

An 85-year-old male was referred from a tertiary hospital with a rapidly growing pearly nodule over the site of a previous BCC excision on the right cheek. An excisional biopsy in the referring centre demonstrated MCC. A wider excision of 1 cm was performed to muscle, and the defect was closed primarily. The patient died from unrelated causes at 6 months post-operative.

### Case 11

An 84-year-old gentleman presented with a slow-growing ulcerating tumour on the vertex of his scalp. An excisional biopsy confirmed the diagnosis of MCC without perineural or lymphovascular invasion. A wider excision of 2 cm was performed, and the defect was resurfaced with a local transposition flap. A CT brain and PET CT were negative for metastatic disease. He is currently awaiting radiotherapy to the tumour site.

## Discussion

The incidence of head and neck MCC has steadily risen over the last decade.^13^ As the Western population ages, its incremental increase is above that of melanoma and all solid tumours.^10^^,^^13^ The head and neck are the most common location of a primary MCC.^3^^,^^10^^,^^14^^,^^15^ The head and neck MCC has the propensity for loco-regional recurrence, and early microscopic spread to regional nodal basins, and distant metastasis which makes it challenging to treat.^17^ Reported rates of disease-associated 5-year mortality are as high as 46%.^16^ The presence of conflicting staging systems has complicated the management of MCC, and the single most important measure of recurrence and metastasis is the stage at diagnosis.^12^ However, treatment guidelines are not well defined, predominantly due to the rarity of the tumour, which prohibits clinical trials.^15^ Nonetheless, the mainstay of head and neck MCC management is dependent on accurate histopathological interpretation, micro-staging of the primary lesion, surgery, and radiotherapy.^4^

### Surgical Management

Early wide excision remains the primary treatment of MCC.^4^ There are no randomized controlled trials of excision margins and disease-specific control.^10^^,^^15^^,^^18^ Excision to fascia with a negative lateral margin of at least 2 to 3 cm is preferred.^4^^,^^15^ However, previous studies have shown little effect on recurrence-free survival with wider margins.^10^ Obtaining wide clear margins can be challenging in the head and neck with cosmetic and functional impairment.^16^ As this tumour has a tendency to extensive vertical growth, some advocate the use of Mohs micrographic surgery.^18^ The benefits of Mohs include the preservation of normal tissues of important anatomical regions.^19^ However, head and neck trial numbers including Mohs excision remain low.^10^^,^^11^^,^^20^ O'Connor et al. found Mohs superior in local control to standard surgical excision, but suggested the adjunct use of radiotherapy in these patients.^19^ MCC has been shown to spread in a non-contiguous manner, and the risk of recurrence post-Mohs surgery is substantially higher if no radiotherapy is delivered.^19^

In our case series, there was no significant survival benefit demonstrated with wider surgical margins. This finding is demonstrated by other authors’ experience with head and neck MCC.^10^^,^^11^^,^^18^^,^^21^ Unfortunately, many case series to date have not reported data on margin and survival benefit.^3^^,^^22^ Surgery alone was used in several of our cases. It has been well documented that surgery alone can be sufficient in low-risk MCC tumours.^23^^,^^24^ Radiotherapy may not be needed if the tumour is less than 2 cm in size, wide excision margins have been achieved, and no other high-risk histological features.^23^^,^^24^ However, if any of the high-risk factors are present, selective adjuvant radiotherapy should be considered.^4^^,^^23^^,^^24^

### Radiotherapy

The NCCN *Clinical Practice Guidelines in Oncology* for MCC recommends selective use of adjuvant local radiotherapy, and its definite use in all nodal disease.^4^ However, no level one evidence currently guides this application.^4^^,^^23^ In the largest cohort study to date, Bhatia *et al.* demonstrated an overall survival benefit with surgery combined with adjuvant radiotherapy in stage I-II MCC.^25^ Similarly, Lewis *et al*. report an increase overall survival in all stages with surgery combined with radiotherapy.^26^ These findings have been questioned by smaller cohort studies, advocating selective use in high-risk tumours.^23^^,^^24^ Fields *et al.* examined the pattern of recurrence in 364 patients who underwent surgery with or without adjuvant treatment for stages I through III MCC.^23^ Patients with stage I-IIIA MCC and clinically negative lymph nodes had a low recurrence rate with adequate surgery and selective use of adjuvant radiotherapy for high-risk tumours.^23^ On the other hand, patients with clinically positive lymph nodes and stage IIIB MCC had significantly higher recurrence rates with the same treatment.^23^

Assessing the value of adjuvant radiotherapy specifically in the head and neck clinical context remains difficult.^4^ Specific retrospective evaluation of the benefit of radiotherapy in head and neck MCC has been demontrated.^10^^,^^27^^,^^28^ Clark *et al*. report that combination therapy was associated with improvement in local and regional control and disease-free survival in stage II and III MCC of the head and neck.^27^ Similarly, Veness *et al*. substantiate its use in all stages of head and neck disease, with high rates of locoregional relapse in its absence.^28^ This trend was comparably demonstrated in smaller reported case series institutional experiences.^10^ Overall, our institutions’ experience supports the NCCN practice guidelines.^4^

### Role of Sentinel Lymph Node Biopsy

MCC of the head and neck has a high propensity to metastasise to the lymph nodes, and recent attention has been focused on the use of SLNB.^29^ Accurate staging is required for appropriate treatment planning and development of clinical trials in head and neck MCC.^3^ It has been demonstrated that approximately one-third of MCC patients who only undergo clinical nodal evaluation are under-staged due to the presence of occult microscopic nodal involvement.^17^ Sentinel node status has a significant prognostication value, with nodal positivity reflecting high rates of recurrence or metastasis.^29^ Conversely, sentinel node negativity may be associated with a significant survival advantage.^30^

The role of sentinel lymph node biopsy may extend with therapeutic benefit in recurrence protection and progression.^4^ Kachare *et al.* report a small, but significant, DSS in patients who underwent SLNB compared to those who opted for nodal observation only.^30^ Similarly, Kaae *et al.* report a survival benefit in the SLNB arm of a retrospective review in comparison with those who did not have an SLNB.^31^ However, the prognostic significance of sentinel node status may be different in head and neck MCC compared with other anatomical sites. Lentsch *et al*. report no predictive survival benefit with sentinel node status in MCC specific to the head and neck.^3^ However, these findings should be interpreted with caution due to the limitations of their recurrence data.^3^

Our institutions’ experience of SLNB in head and neck MCC reflects the tumours rarity. Unfortunately, reported case series of head and neck MCC are similar.^10^^,^^11^^,^^14^^,^^22^^,^^32^^,^^33^ However, the overall trend demonstrates SLNB as a safe and reliable technique for the staging of MCC of the head and neck. Therefore, as per the NCCN guideline, SLNB should be recommended for all patients with clinically node-negative head and neck MCC who are fit for surgery.^4^

### Systemic Therapy

High-quality clinical data on the delivery of post-operative systemic agents for head and neck MCC is lacking.^4^ The majority of available data is pooled from retrospective reviews of a variety of stages, concomitant therapies, anatomical locations, and different systemic agents. In the largest review to date, Chen *et al.* concluded that chemoradiation increased overall survivability in addition to surgery, but chemotherapy alone had the opposite effect.^34^ This suggests that although chemotherapy as a post-operative monotherapy is likely to be unsuccessful, there may be a role for chemoradiotherapy in high-risk cases. Due to the lack of evidence for increased survival, associated morbidity, and rapid development of resistance, its routine use remains unsupported.^35^^,^^36^ Our own institutions most common systemic treatment of metastatic or palliative disease is cisplatin or carboplatin with or without etoposide, which is consistent with current conventions.^4^^,^^11^^,^^15^^,^^33^^,^^37^ However, no established treatment based on validated evidence has been determined to date.^5^

Emerging data on targeted therapies for MCC is promising.^16^ The PD-1 antibody pembrolizumab and the PD-L1 antibody avelumab have been shown to induce partial and complete remission in advanced MCC.^38^ Both virus-positive and virus-negative tumours were shown to be immunogenic and susceptible to therapy. Ongoing trails continue to support the success of avelumab monotherapy in patients with distant MCC.^39^^,^^40^ These results suggest that immune checkpoint inhibitors may have a role in the first-line management of advanced disease. Although there are no randomized comparative trials that demonstrate the superiority of checkpoint immunotherapies over chemotherapy, they may provide more longevity in response.^4^

## Conclusions

MCC of the head and neck remains a rare and aggressive disease entity with diagnostic and treatment challenges. The results of our retrospective review should be interpreted with caution due to the limited cohort. However, our case series adds to a sparse body of evidence of head and neck MCC. Surgery remains the treatment priority in localized disease, with an increasing role of SLNB for accurate prognostication and staging. Early management of stage I disease results in moderate long-term disease-free survivability. Radiotherapy is recommended for high-risk tumours. Clear recommendations on the use of systemic therapies are lacking, but prospective trials are promising.
